# Balancing trade-offs in circular medical device design: a case study on laparoscopic devices

**DOI:** 10.1007/s44498-026-00189-8

**Published:** 2026-09-20

**Authors:** Tamara Hoveling, Naomi Muindi, Jeremy Faludi, Conny Bakker

**Affiliations:** 1 Department of Sustainable Design Engineering, Delft University of Technology, Delft, The Netherlands; 2 Department of Green Chemistry and Technology, Faculty of Bioscience Engineering, Ghent University, Ghent, Belgium; 3 Faculty of Industrial Design Engineering, Delft University of Technology, Landbergstraat 15, 2628 CE Delft, The Netherlands

**Keywords:** Industrial ecology, Circular design, Medical devices, Life cycle assessment, Sustainable healthcare, Circular design trade-offs

## Abstract

This study explores how circular design principles can be applied to single-use laparoscopic staplers to improve their carbon footprint without compromising critical requirements such as safety, performance, and financial feasibility. Using a research-through-design approach, four alternative concepts were developed: a fully recyclable device, a partially reusable device, a multifunctional device, and a manual device, aiming to reduce the carbon footprint while maintaining clinical performance of the device. Iterative design cycles incorporated feedback from Medtech professionals and from qualitative surveys with additional industry stakeholders. Fast-track life cycle assessments (LCAs) estimated environmental impact compared to the baseline device, while stakeholder feedback assessed trade-offs and synergies related to safety, usability, workflow compatibility, and regulatory compliance. Here, “trade-offs” refer to improvements in circularity coming with losses in other requirements, while “synergies” improve both circularity and other requirements together. Results revealed that each concept delivered potential carbon footprint reductions, particularly the reusable, multifunctional, and manual variants. Trade-offs included increased sterilization burden, altered surgical workflow, and reduced user familiarity; synergies included lower material and logistics costs from reuse and multifunctional design, achieved without compromising safety or usability. The findings highlight that achieving circularity in medical device design involves more than technical considerations; it requires a systems-level approach. Effective implementation depends on aligning design strategies with regulatory requirements, clinical workflows, and supply chain practices. This study provides recommendations to navigate trade-offs and identify synergies, supporting a structured approach to advancing sustainable practices in healthcare.

**Supplementary Information:**

The online version contains supplementary material available at https://doi.org/10.1007/s44498-026-00189-8.

## Introduction

As the healthcare environmental footprint grows (Karliner et al., 2019; MacNeill et al., 2017), the urgency for sustainable solutions intensifies. Among medical waste sources, single-use devices—such as powered laparoscopic instruments—are particularly concerning due to widespread use, short lifecycles, and embedded electronic components, which add to mounting medical and electronic waste (e-waste) (Keil et al., 2022; Vanderwee et al., 2024; Wombwell et al., 2024). The environmental impact of medical devices is an increasingly important consideration within healthcare sustainability, particularly in the context of electronic waste, which is one of the fastest-growing global waste streams and remains relatively underexamined in this sector (Montesinos et al., 2024).

Laparoscopic surgical instruments—such as long-shafted scissors, clamps, and staplers—are used in minimally invasive surgeries via small 1 to 1.5 cm incisions in the abdomen or pelvis (see visualisation in Supplementary Information SI1 (Appendix A)).

These procedures have improved patient outcomes by reducing blood loss, narcotic use, and hospital stays compared to open surgeries (Nguyen et al., 2007; Shapiro et al., 2011; Shi, 2024). In this way, the emergence of laparoscopic surgery has already contributed to healthcare sustainability. However, the number of laparoscopic procedures is increasing rapidly—over 13 million were performed globally in 2020 (Research, 2020). The growing reliance on single-use and powered laparoscopic devices during those procedures, intended to reduce infection risk, exacerbates environmental challenges, especially through e-waste. Thus, while laparoscopic devices improve health outcomes, their environmental footprint—particularly through increased e-waste, emissions, and resource-intensive production—may contribute to broader public health risks beyond hospital walls.

Fortunately, there are opportunities for improvement. For example, Meissner et al., (2021) demonstrated that switching from single-use to multi-use surgical staplers significantly reduced waste and material requirements—by 40% and 92%, respectively—in laparoscopic sleeve gastrectomy, with similar reductions for other procedures. Similarly, Rizan and Bhutta (2022) reported that hybrid laparoscopic instruments—combining reusable and disposable parts—had a carbon footprint of approximately 1756 g CO_2_ equivalent (CO_2_-eq) per operation, versus 7194 g CO_2_-eq for single-use equivalents, a 75% reduction. It is important to note that these instruments are less technically complex than powered laparoscopic staplers and therefore may not be directly comparable. Nevertheless, these studies demonstrate that circular strategies can substantially reduce the environmental impact of laparoscopic devices and provide evidence that even more complex instruments may warrant exploration for circular redesign. Given their material intensity and reliance on electronics, powered laparoscopic staplers represent a challenging but potentially impactful opportunity for further investigation.

Most laparoscopic instruments—including powered staplers—are however single-use. They are disposed after each procedure, sometimes after each actuation, meaning multiple staplers or their components may be disposed of in a single surgical procedure, leading to waste of valuable materials and electronic components. This continued reliance on single-use devices is largely due to the challenges involved in adopting a circular model. These challenges include maintaining clinical safety and performance while supporting sterilization and reuse, navigating regulatory frameworks, managing electronic waste, minimizing workflow disruptions, and addressing concerns about reliability and user acceptance (Hinrichs-Krapels et al., 2022; Hoveling et al., 2024; MacNeill et al., 2020). Research exploring these trade-offs, especially for powered laparoscopic devices, remains limited. Although circular economy principles—such as reuse, refurbishment, recycling—offer promising paths to reduce resource use and waste in healthcare (Ellen MacArthur Foundation, 2019), their adoption in medical technology is still in early stages.

Existing circular design literature highlights trade-offs among cost, safety, clinical performance, and environmental impact in medical device procurement and use (Burton et al., 2021; Chauvet et al., 2024; Costa et al., 2018). Yet few studies directly address common design challenges in circular medical devices. Prendeville et al. (2017), for example, examined eco-design dilemmas across industries, identifying tensions and contradictions in balancing sustainability with other requirements. Their framework offers operational, tactical, and strategic responses but does not focus on healthcare’s unique demands. Likewise, Samenjo et al., (2023) did focus explicitly on healthcare-setting product design—evaluating circular economy implementation in low-resource contexts via the Chloe SED® device—but their insights are confined to developing markets, offering limited guidance for high-income, highly regulated environments such as the EU and US.

This study explores single-use powered laparoscopic staplers as a case study to explore how circular design can be applied in practice under the strict safety and regulatory constraints of healthcare systems. It addresses the following research question: *How can trade-offs and synergies between circularity, safety, functionality, usability, and regulatory compliance be systematically assessed and managed in the design of complex medical devices?* Focusing on this device category allows the study to examine the interactions between environmental goals and the non-negotiable requirements of healthcare. Research-through-design (RtD) methods were used to develop and iteratively refine four concepts—fully recyclable, partially reusable, multifunctional, and manual—while fast-track life cycle assessments (LCAs) quantified environmental impact and stakeholder feedback captured trade-offs and synergies. By integrating RtD, fast-track LCA, and stakeholder input, the study provides practical recommendations for advancing circular medical devices without compromising clinical outcomes or market viability.

## Methods

This study followed a circularity-driven research-through-design (RtD) approach (Zimmerman et al., 2007) to redesign a medical product for improved circularity and a lower carbon footprint. RtD is a methodology in which design activities themselves are treated as a way of learning: creating prototypes and iterating on concepts generates knowledge about complex systems, user needs, and real-world constraints.

The baseline device—a powered linear laparoscopic stapler commonly used for transecting and sealing tissue—was first analysed to understand material composition, assembly complexity, and opportunities for reuse, recycling, or simplification. Observations of the device in use within the operating room and sterilization department, together with consultations with surgeons and engineers conducted during these observations, helped capture practical handling challenges and workflow considerations. These observations were summarized in a simplified product journey map (simplified version in Supplementary Information SI1 (Appendix B), which documents the device’s lifecycle, handling steps, and system-level opportunities for improvement.

**Fig. 1:**
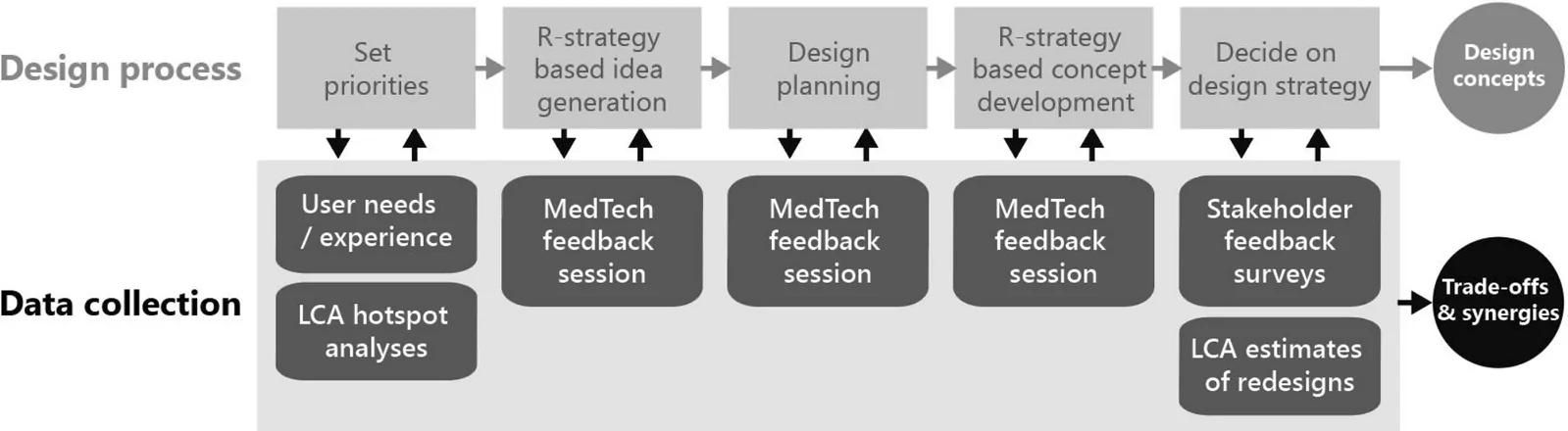
Overview of RtD methodology

Four design concepts were then developed through iterative cycles, guided by expert input from a Medtech firm and other stakeholders (Fig. 1). The aim of these RtD iterations was to explore and compare alternative circular design interventions and identify associated trade-offs, rather than to converge toward a final optimized product concept. The design process followed circular design guidelines adapted from (Hoveling et al., 2024), alongside additional guidelines addressing regulatory, usability, and safety requirements. Regulatory requirements were integrated through expert consultation (Supplementary Information SI1 (Appendix C). Environmental hotspot analysis (fast-track LCA) was used separately to identify priority areas for improvement in this specific device and to provide a baseline for comparison with the redesigned concepts.

The RtD approach allowed identification of opportunities to improve circularity and lower the carbon footprint, highlighted areas where compromises were needed, and made trade-offs explicit, providing insights beyond this specific stapler. Concepts were developed using a morphological chart, a design tool that breaks a product down into its main subfunctions and explores alternative solutions for each (Boeijen et al., n.d.). For this study, the approach was adapted to consider circular strategies—such as refuse, reuse, repair, and recycle—rather than only functional decomposition. This made it possible to systematically identify design opportunities that boost circularity while still meeting clinical and regulatory requirements. Concepts were refined through structured feedback, with updates to the circular design guidelines after each cycle (final version in Supplementary Information SI1 (Appendix D).

Trade-offs between circularity and other key design requirements, such as safety, functionality, and economic feasibility, were evaluated through (1) the level of circularity improvement per concept, (2) expert feedback, (3) stakeholder surveys, and (4) challenges identified during the design process. In this context, trade-offs are defined as the ways improvements in circular design objectives (e.g., durability, reparability, or recyclability) are balanced against drawbacks in one or more performance domains—such as safety, usability, workflow integration, or regulatory compliance. As the devices assessed in the LCA were conceptual, expert feedback and stakeholder survey responses were based on projected performance and assumptions, rather than on validated real-world devices.

### Life cycle assessment

Fast-track LCAs were conducted to (1) set priorities for redesign from environmental hotspots; (2) decide between design concepts by comparing their estimated LCA performance to the baseline; and (3) uncover challenges in designing for circularity, highlighting additional trade-offs or synergies.

The fast-track LCAs were performed on the product level; although full-system LCAs provide broader insights into healthcare pathways (Muindi et al., 2025), a product-level approach was chosen for its practicality and focus on redesign-relevant impacts. The study followed a cradle-to-grave system boundary, covering raw material extraction, manufacturing, packaging, transportation, use, and end-of-life (EoL) treatment. Assembly and gamma sterilization prior to distribution were excluded due to their expected minor contribution based on previous assessments of similar medical devices. The assessment was performed using SimaPro version 9.5.0.1, with background data sourced from Ecoinvent v3.9.1. The Environmental Footprint (EF) 3.0 method was used for Life Cycle Impact Assessment (LCIA). The functional unit was defined as the environmental impacts per use cycle. This reflects the clinical service delivered rather than the product itself, thereby enabling a fair comparison between single-use and (partially) reusable device concepts.

Finally, both the baseline and circular design concepts were assessed for 50 (re)use cycles, respectively, based on manufacturer guidelines specifying up to 50 uses with proper cleaning and sterilization (*Laparoscopy by Ackermann*, n.d.). Since actual reuse performance depends on factors such as device durability, maintenance, and clinical validation, this parameter represents and assumption for comparative modelling rather than a definitive prediction of achievable reuse cycles. Because reuse cycles and sterilization requirements strongly influence the environmental performance of reusable concepts, these parameters should be considered key uncertainty drivers. However, due to the conceptual nature of the designs and the absence of validated device performance data, a quantitative sensitivity analysis was beyond the scope of this exploratory study. Future validation studies should specifically assess sensitivity to reuse frequency, sterilization intensity, and maintenance requirements.

For end-of-life modelling, system expansion was applied as the allocation method, crediting the system for energy recovery from incineration and recyclate substitution in the case of recycling, based on a 1:1 substitution assumption. The baseline product and use scenario was a powered linear laparoscopic stapler; a single-use device used for transecting and sealing tissue during minimally invasive surgical procedures, which is incinerated after use. This device served as the starting point for the subsequent redesign within the research-through-design (RtD) process. The baseline product model was constructed using primary data provided by the device manufacturer, including information on materials, production processes, transport, packaging, and end-of-life characteristics. Secondary data from peer-reviewed literature and the ecoinvent database supplemented missing information where necessary. To model the four circular design concepts, the baseline data were updated to reflect key changes such as alternative materials, reuse potential, and sterilization requirements, while maintaining consistent system boundaries and usage context, with larger uncertainties assigned to aspects with less clarity or more variation (e.g., number of reuses). Due to limited availability of detailed manufacturer-specific data, several parameters were based on available guidelines, literature, and design assumptions. These uncertainties should be considered when interpreting the results. For the purpose of the LCA, the device was assumed to be assembled in the US, and used in a Belgian hospital, and Belgium-specific EoL treatment was applied accordingly. Surgical staples were excluded from the model, as staple type and quantity vary between procedures and could not be standardized across the use cases.

### Identifying and analysing trade-offs

Trade-offs between circularity benefits and challenges were identified through an analysis of stakeholder surveys and expert (Medtech) feedback on the product redesigns and were further informed by the design guidelines developed earlier. These guidelines were based on environmental hotspot analysis (fast-track LCA), consultations with surgeons and engineers, and observations in the OR and sterilization department. In this study, trade-offs were defined as situations where an intervention intended to improve circularity or reduce carbon footprint resulted in potential challenges for other critical medical device requirements, including safety, functionality, usability, regulatory compliance, technical feasibility, or financial viability. Synergies were defined as situations where a circular design intervention was expected to improve environmental performance while simultaneously providing benefits in one or more of these additional requirements. The insights were examined using both qualitative and quantitative approaches to compare the environmental improvements of the circular designs against challenges related to financial viability, logistics, and technical feasibility. To integrate the environmental and stakeholder-based evaluations, an alignment assessment was performed by comparing the relative environmental performance of each concept from the LCA with stakeholder and Medtech evaluations across the identified design criteria. Concepts showing high environmental potential but lower perceived feasibility were interpreted as cases where implementation barriers may limit adoption, whereas concepts showing moderate environmental benefits combined with higher acceptance were considered potential transitional pathways. This comparison was used to identify mismatches between measured environmental performance and stakeholder perceptions, rather than to establish an overall scoring of the concepts. Identified trade-offs and synergies were subsequently categorized according to the underlying circular design intervention (e.g. enabling reuse, reducing electronics, multifunctional design, or improving recyclability) to identify recurring patters across concepts. This approach enabled actionable recommendations that balance circularity gains with other key design considerations.

**Fig. 2:**
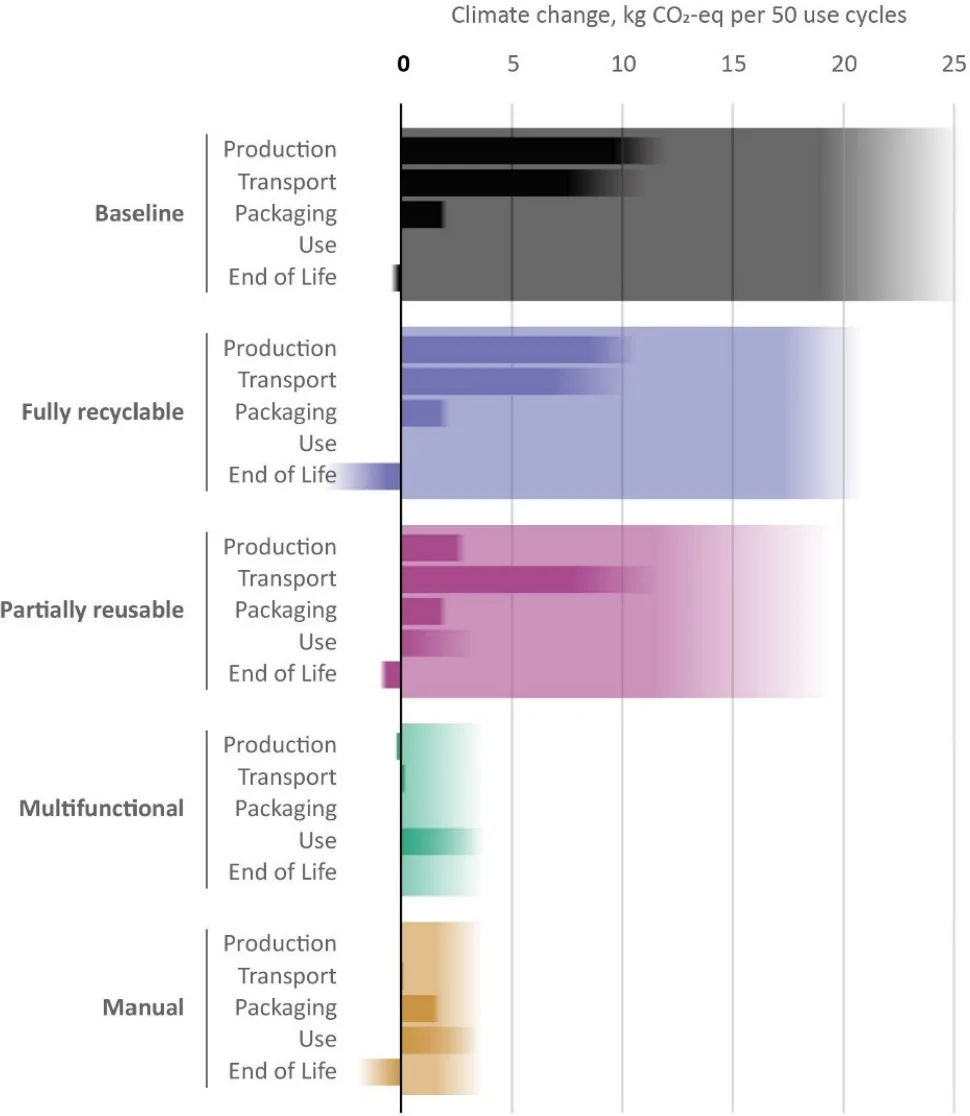
Climate change in kg CO2-eq. per 50 use cycles for total impacts of the baseline versus new design concepts, including climate change per process. Gradients represent uncertainty. The underlying LCA data used to generate this figure are not publicly available due to third-party confidentiality restrictions; see the Data Availability Statement

A phased approach (as displayed in Fig. 2), starting with Medtech feedback before involving other stakeholders, was adopted for several reasons. First, Medtech professionals bring specialized expertise in regulatory compliance, medical safety, and technical feasibility, ensuring that designs meet essential industry standards early on. Second, this strategy allows for focused, efficient refinement, preventing premature exposure of underdeveloped ideas to a broader audience. Resolving significant technical and functional challenges early ensures that feedback from other stakeholders can focus on broader considerations such as user experience, sustainability, and market acceptance. Lastly, this phased approach prevents feedback overload, guiding early design decisions with expert input while reserving diverse perspectives for later stages.

#### Medtech feedback sessions

At the end of each iterative design cycle, design concepts were evaluated in three online feedback sessions with key stakeholders from a large Medtech firm, representing environmental sustainability, global sustainability oversight, and R&D product leadership in surgical stapling. These sessions aimed to identify potential trade-offs associated with circular design interventions and re-evaluate design decisions to inform subsequent iterations. Feedback sessions were scheduled at critical points to ensure insights could be integrated in the next design cycle. Participants evaluated design ideas from both product-specific and system-wide perspectives:

Product-related aspects:

- *Functionality and performance*: how design changes affect the device’s ability to meet user and patient needs.
- *Ease of use and maintenance*: implications for usability and long-term maintenance.
- *Medical safety*: risks related to contamination, perceived safety, and adherence to safety protocols.

System and implementation aspects:

- *Financial viability*: feasibility from a business and market perspective.
- *Regulatory compliance*: potential challenges in meeting medical and non-medical standards.
- *Technical feasibility*: the practicality of implementing the design using current technologies.

A primary focus was on identifying the trade-offs associated with circularity, with participants reflecting on how design decisions could reduce the carbon footprint across the product lifecycle, while potentially jeopardizing design requirements in other areas, such as functionality, safety, financial viability, or regulatory compliance. The Medtech feedback sessions were recorded and summarized. These meetings captured expert observations grouped under the key evaluation perspectives (e.g., environmental sustainability, functionality, financial viability).

The Medtech experts jointly discussed and assigned a consensus score for each concept and criterion using a 1 to 4 ordinal scale, where 1 represented the lowest perceived performance. A 1–4 scale was selected to encourage differentiation between concepts and avoid a neutral midpoint, as the objective was comparative scoring rather than measuring absolute agreement. Because the scores were generated through group discussed rather than individual questionnaires, the results present consensus scores rather than averaged individual ratings. Heatmaps were generated to visualize those expert consensus scores. Additionally, the experts were requested to explain the reasoning behind their scores, reflecting on how specific design choices affected the different perspectives. Trade-offs were identified by examining cases where concepts with a higher carbon footprint were scored higher than those with a lower carbon footprint, and vice versa. When a concept scored low on certain aspects despite offering more circularity interventions, we analysed the underlying reasons for these discrepancies.

#### Stakeholder surveys

Following the development of four design concepts, a broader group of stakeholders (N = 10; participant characteristics are provided in Supplementary Information SI1 (Appendix E) was invited to assess the designs. This qualitative evaluation involved stakeholders scoring each concept on a Likert scale ranging from “very poor” to “very good,” with “acceptable” as the neutral midpoint, for the same evaluation perspectives as were discussed with the Medtech experts above. Participants also provided written explanations for their scoring, highlighting trade-offs or synergies associated with the circular design in terms of functionality, sustainability, financial viability, and other factors. A full version of the survey is provided in Supplementary Information SI1 (Appendix F).

Although some participants lacked direct healthcare expertise, their inclusion was intentional. As circularity is still emerging in healthcare design, experts from sustainability and circular business fields offered valuable, cross-industry insights. Their broader perspective helped identify design trade-offs that may be overlooked by those within the healthcare sector.

To enable a structured comparison of the design concepts, stakeholder survey data were analyzed using the same evaluation criteria as those applied in the Medtech consensus scoring. The stakeholder survey (N = 10) used a 1–5 Likert scale across seven criteria: financial viability, logistical feasibility, functionality and performance, ease of use and maintenance, regulatory compliance, medical safety, and technical feasibility. For each concept–criterion combination, the mean score across participants was calculated, resulting in 28 aggregated values (four concepts × seven criteria). The resulting mean scores ranged from 3.0 to 4.9, with no scores falling below the neutral midpoint of the Likert scale. Because stakeholder evaluations were generally positive and clustered within the upper part of the scale, direct visualization using the original Likert range would have reduced the ability to distinguish relative differences between concepts. Therefore, the 28 aggregated values were normalized to a 0–1 scale using min–max normalization: *(x − 3.0)/(4.9 − 3.0)*. This transformation was applied solely to improve visual discrimination in the heatmap and does not imply equivalence between the stakeholder survey and Medtech expert scoring approaches. The normalized scores were visualized using quartile-based colour coding to facilitate comparison of relative performance patterns between concepts. The normalized scores were used only for the visualization of the results. The subsequent interpretation of stakeholder preferences, trade-offs, and synergies was based on the individual survey responses and accompanying qualitative explanations.

## Results

The summarized results of the baseline fast-track LCA (assessing the single-use linear powered stapler) are presented in Supplementary Information SI1 (Appendix G). While the manufacturing impacts seem to dominate overall, they are not significantly different from transport impacts. Transport impacts were high, because different processes (e.g. assembly, sterilization, use, etc.) are based in different regions (or even countries) due to which a lot of transport is needed. The geographical scope was based on a representative European healthcare setting, with Belgium selected as the nominal location of device use. This assumption was used to define transport routes and does not represent a specific clinical or market context. This includes for example assumed air transport from the distribution centre in the US to hospitals in Belgium. Based on the LCA, key environmental hotspots were identified, particularly in the production of printed circuit boards and batteries. Usage impacts are zero because the device’s battery is already charged in production, no additional charging is performed in the surgery theatre. EOL impacts are net negative because the total treatment burden is fully offset by credit from the energy recovered during the incineration of hazardous waste.

The fast-track LCA hotspots, combined with the established design guidelines (Supplementary Information SI1 (Appendix D), highlighted challenges for improving environmental sustainability at both the product and system levels. System-level considerations—such as material separation, component reuse, and handling—further shaped the design concepts. These insights guided the iterative redesign process. Four conceptual redesigns were developed to enhance circularity while addressing potential trade-offs. Each concept—refined through iterative expert feedback—targets specific hotspots using distinct circular strategies, as summarized in Table 1.

**Table 1:** Design concepts and their addressed hotspots

Design concept	Description	Addressed hotspots/challenges
Recyclable device	Hospital separates battery, decontaminates components, and sends both battery and device for recycling. Compact design with three click-separable compartments: metal shaft, plastic casing, electronics with single-use battery. Clear icons guide disassembly	∙ Material recovery at end-of-life ∙ Battery management simplified through separable electronics unit ∙ Efficient separation workflow
Partially reusable device	Internal electronics unit is reusable; outer components are single-use and recyclable. Electronics unit is removed, recharged, and reused; plastic casing and other parts are recycled. After ~50 uses, electronics are recycled	∙ E-waste reduction through electronics reuse ∙ Battery charging ∙ Risk of battery contamination (in case of battery reuse) minimized ∙ Workflow integration for disassembly and recharging
Multifunctional device	Modular device with reusable main unit and interchangeable click-on shafts. Electronics housed in main unit; shafts and shell are swapped and sterilized after each use. Tracking system monitors usage; electronics refurbished or recycled after lifespan	∙ Electronics consolidation (minimizing need to use multiple electronic devices) ∙ Material efficiency ∙ Reuse tracking ∙ Operational flexibility across procedures
Manual device	Fully manual device with no electronics. Designed for full reuse for as far as appropriate, cleaned and sterilized after each use. Refurbished at end-of-life, then recycled if necessary. Focus on durability and simplicity	∙ Completely eliminates electronics and battery management ∙ Maximizes ease of reuse ∙ Simplified workflow and sterilization

Simplified system maps and concept illustrations can be found in the stakeholder surveys provided in Supplementary Information SI1 (Appendix F).

### Life cycle assessment (LCA) comparison

The baseline product and four design concepts were evaluated using LCA to compare their carbon footprints. The environmental impacts of each design concept were derived by modifying the baseline LCA data to reflect concept-specific design changes. The assumptions underlying these modifications are provided in Supplementary Information SI1 (Appendix H).

As Fig. 2 shows, the multifunctional device and manual Device demonstrated the likely lowest impacts in kg CO_2_-eq. This was achieved either by eliminating electronic components or by consolidating multiple functions into a single reusable electronics module with separate metal actuator modules—thereby avoiding most of the carbon footprint (kg CO_2_-eq) associated with the production, use, and disposal of several separate products. The Partially Reusable Device offered a moderate improvement from baseline by extending the lifespan of electronic components and reducing electronic waste, though it still incurred carbon footprint impacts (kg CO_2_-eq) related to electronics. The Recyclable Device offered the least improvement in in life-cycle carbon footprint (kg CO_2_-eq). Despite being designed for disassembly and disintegration in shredding for material recovery, its single-use nature meant its impacts were almost the same as baseline, because most of the device’s total life cycle carbon footprint impacts are from manufacturing the electronic components. As expected, the reusable design concepts scored higher in terms of reduced life cycle carbon footprint. However, the impact of the use phase increased due to the need for disinfection, sterilization, and battery charging. Notably, this increase did not dominate the overall life cycle results or create a new environmental hotspot.

### Assessment of product- and system-perspectives

The comparative assessments of the four device concepts, based on stakeholder survey data and Medtech expert evaluation, are summarized below. Table 2 presents a heat map of the Medtech expect consensus scores obtained through group discussion, where red (1) indicates lower/poorer score, orange (2) indicates intermediate/less favorable values, yellow (3) represents moderately good performance, and green (4) indicates higher/better score. These scores represent the collective judgment of the Medtech experts rather than average individual ratings. Further clarification of how the scoring was performed can be found in the methods description (Sect. 2.2.1).

**Table 2:** Medtech expert consensus scores (Raw 1–4 scale)

Average score of …	Recyclable design	Reusable design	Multifunction design	Manual design
Financial viability	1	2	3	4
Logistic feasibility	4	2	3	1
Functionality & performance	4	2	1	3
Ease of use & maintenance	4	2	1	3
Regulatory compliance	4	1	2	3
Medical safety	4	2	1	3
Technical feasibility	4	3	2	1

These consensus scores provide an industry-focused perspective on perceived feasibility and implementation challenges, complementing the broader stakeholder evaluation. Table 3 presents the aggregated stakeholder survey results using the same evaluation criteria as the Medtech assessment. Unlike the Medtech experts, who scored concepts on a forced 1–4 scale, stakeholders evaluated each concept independently using a 1–5 Likert scale. The mean score was calculated for each concept–criterion combination, resulting in 28 aggregated values. Because these mean scores were generally high and clustered between 3.0 and 4.9, they were normalized to improve visual discrimination before applying quartile-based color coding (see Sect. 2.2.2). The resulting heat map therefore illustrates relative differences between concepts rather than absolute performance levels. The detailed concept discussions presented in later sections additionally draw on participants' individual concept preferences and qualitative explanations, which are reported separately from these aggregated quantitative results.

**Table 3:** Relative, normalized survey scores (Normalized over all 28 values; quartiles: ≤ 0.3575; > 0.3575–0.525; > 0.525–0.68; > 0.68)

Average score of …	Recyclable design	Reusable design	Multifunction design	Manual design
Financial viability	0.16	0.26	0.32	0.47
Logistic feasibility	0.37	0.42	0.00	0.63
Functionality & performance	0.68	0.74	0.63	0.11
Ease of use & maintenance	0.63	0.58	0.11	0.42
Regulatory compliance	0.74	0.63	0.37	0.68
Medical safety	1.00	0.74	0.47	0.47
Technical feasibility	0.68	0.68	0.11	0.58

Table 4 provides a structured overview of the relationship between estimated environmental performance and stakeholder evaluations, highlighting where sustainability potential aligns with perceived feasibility and where mismatches occur.

**Table 4:** Integrated comparison of environmental performance and stakeholder perception

Design concept	LCA environmental potential	Stakeholder/expert perception	Main interpretation
Recyclable device	Limited improvement	High acceptance	Easy integration into current workflows, but limited environmental benefit because the main hotspots remain unchanged
Partially reusable device	Moderate improvement	Medium to high acceptance	Provides a balance between environmental improvement and perceived feasibility
Multifunctional device	Medium to high improvement	Low perceived feasibility	High environmental potential but requires further validation of technical feasibility, usability, and workflow integration
Manual device	Highest improvement	Mixed perception	Strong environmental potential and simplicity benefits, but concerns remain regarding clinical performance and technical feasibility

Because stakeholder scorings were generally favorable across concepts, differences between concepts should be interpreted as relative differences in perceived feasibility and desirability rather than as clear acceptance or rejection of individual designs. However, the integrated comparison still revealed that environmental performance and stakeholder preference did not always align. For example, the Recyclable Device was perceived as highly feasible by stakeholders despite offering limited carbon reduction, whereas the Multifunctional and Manual concepts demonstrated greater environmental potential but faced higher perceived implementation barriers. These mismatches indicate that adoption decisions are influenced not only by environmental performance but also by familiarity, perceived risk, and compatibility with existing healthcare systems.

#### Recyclable device

Table 2 shows that the Medtech experts’ consensus evaluation identified the Recyclable Device as the overall preferred concept, despite assigning it a low score on financial viability. This aligns with Table 3, where the concept scores relatively high in regulatory compliance (0.74) and medical safety (1.00), reflecting its perceived reliability and familiarity. They valued its familiar form and minimal perceived disruption to clinical workflows. Stakeholder survey respondents confirmed its ease of integration, noting that it seems “the device does not require maintenance,” “the collection of the devices is done as a unit, which is easier to handle,” and “most of the infrastructure for the value chain should already be in place.” They also highlighted its safety benefits: “less contamination and malfunction risks than reusing or refurbishing,” and “safety is guaranteed with no risk of cross-contamination.” Yet the same respondents rejected it for its environmental shortcomings, warning that “the recycling of plastic is currently limited because of the cost of virgin plastics being much lower”. They further cautioned that reliance on virgin feedstocks could incur “significant costs for transportation and use of those virgin materials,” underscoring the trade-off between immediate feasibility and long-term sustainability.

#### Partially reusable device

The Partially Reusable Device scored second-worst in LCA impact but was the second-most preferred overall, as indicated by its mid-to-high normalized scores in Table 3 (e.g., functionality & performance = 0.74; regulatory compliance = 0.63), with 50 percent of stakeholder survey respondents naming it their favourite. Survey participants praised its ability to reduce e-waste while keeping design and logistics largely unchanged: “staff only need to charge the battery as an extra step,” and “as the housing is still single-use, it would probably comply.” In Medtech feedback sessions (Table 2), experts cautioned that the extra steps for charging and reassembly could introduce risks around maintenance, reliability, and aseptic transfer0F,^1^ potentially leading to user resistance. Survey respondents echoed operational concerns—“reusing contaminated medical components/devices/parts presents a challenge in terms of compliance with the regulatory framework,” and “the re-use of ‘contaminated’ components/products involves decontamination and quality control measures, which in turn increases the contamination risk and device malfunction”—even though, in practice, the electronics module never contacts bodily fluids.

#### Multifunctional device

The Multifunctional Device, despite its strong low carbon footprint in the LCA, faced significant concerns regarding its practical implementation, as reflected by its low technical-feasibility and ease-of-use scores in Table 3 and its low expert scoring in Table 2. During the Medtech expert discussions, participants said it was not technically feasible due to differences in e.g. circuitry and actuation, while also highlighting challenges in maintenance, usability, and sterilization. Stakeholders also pointed to logistical complexities related to managing the modular components and their sterilization processes, noting that these “may require additional oversight” and that the “many individual components and their processes make it more complex to use and maintain.” Inventory control was also emphasized as a critical issue, with difficulties in tracking components leading to procurement inefficiencies and the need for specialized routing between departments. Financially, while some recognized potential long-term cost savings through reuse, others expressed concern over “higher initial costs” driven by the complexity of maintenance, training, and multi-function design. Regulatory compliance was frequently mentioned as a key hurdle, especially around “reusing ‘contaminated’ medical components” that demand rigorous decontamination and quality control, increasing both contamination risk and operational complexity. Despite these concerns, the device’s modular adaptability received praise for “allowing multiple surgical uses” and increasing overall functionality. Interestingly, based on the qualitative survey responses, while many participants questioned the practical usability of this device for the user, the surgeon participant explicitly identified it as his preferred concept and cited its usability and practicality as the primary reasons for this preference. This may indicate a trade-off between perceived and real-world operability, emphasizing the need to include clinical end-users throughout the design process. Beyond traditional usability evaluations, clinicians can provide valuable input during early concept development by identifying workflow implications, practical barriers to adoption, and opportunities for circular strategies to be integrated without compromising clinical performance or safety.

#### Manual device

As reflected in the LCA outcomes, both Medtech experts and other stakeholders recognized the manual device as the device with the lowest carbon footprint in theory. However, as Table 2 shows, Medtech experts also described it as the least feasible option. They argued that powered staplers, which exist for ergonomic and performance reasons, have clear benefits over manual staplers. Therefore, reverting to a manual design could compromise usability and surgical outcomes. While reducing electronic components may lower the carbon footprint, experts cautioned that achieving comparable precision and consistency through mechanical means would be highly challenging. If development proceeds in this direction, the focus should be on identifying which electronic functions are essential and exploring smart mechanical alternatives that maintain performance without sacrificing usability.

Other stakeholders echoed these concerns in Table 3, noting that “manual operation may not be ideal for all surgical applications,” and that “electronics have simplified procedures by reducing operation times and improving results and patient care.” They also highlighted higher risks if the device lacks automated checks to ensure correct procedures, placing greater responsibility on operators. Despite these potential downsides, stakeholders identified several synergies of removing electronics. For example, logistics become “simpler, with no complex waste management required,” and “the reuse of components would align with current and upcoming [sustainability-stimulating] regulations.” The device “seems easy to use, with no need for charging batteries or additional steps.” Additionally, some stakeholders suggested that a manual device “could be less costly and thus less profitable”. This highlights the importance of the business model: hospitals, manufacturers, or both could capture value from new pricing strategies. In particular, “profits can likely be increased if you can charge per surgery or per- use” (although this is harder to implement without electronics to track usage). This pay-per-use approach creates new opportunities for revenue sharing and aligns incentives across stakeholders, but it requires reliable usage tracking to be feasible. However, the Medtech experts pointed out that, due to potentially increasing surgical complications, the whole patient journey could become more expensive and have an increased carbon footprint as the treatment of complications is included in the delivery of care.

### Circular design trade-offs

Through the RtD process, we identified a comprehensive set of trade-offs associated with interventions aimed at improving the circularity and lowering the carbon footprint of single-use laparoscopic staplers. The trade-offs were consolidated across all design concepts and represent recurring issues that emerged during the development and evaluation process. Rather than treating individual trade-offs as isolated issues, they were organized into a typology based on the underlying circular design intervention (e.g., enabling reuse, reducing electronics, multifunctional design, and improving traceability). This structure highlights how different sustainability strategies introduce distinct challenges and opportunities, while also revealing recurring themes that may be relevant beyond the specific case study. Table 5 presents the detailed trade-offs within each intervention category, alongside potential strategies explored to manage these challenges.

**Table 5:** Overview of trade-offs per sustainability intervention, with potential solutions

Sustainability intervention: use of recycled materials in production	
Trade-off	Potential ways to manage tension
Potential decrease in quality and performance for needed functionality	Develop high-performance recyclable materials and testing to ensure recycled materials meet quality standards for expected number of use cycles
Increased costs for production, as virgin plastics may be cheaper	Highlight the long-term added value of reducing the dependence on virgin materials
Regulatory compliance: use of recycled materials might be restricted	Use recycled content for non-sterile product parts, and when recycling practices improve, engage with regulators early to update guidelines

Sustainability intervention: enabling safe reuse (incl. sterilization)	
Trade-off	Potential ways to manage tension
Increased hospital costs due to new systems for component maintenance	Shift focus to long-term savings or introduce alternative models (e.g., pay per use)
Logistic complexity and difficulties; need for changes in infrastructure	Utilize logistics systems of e.g. sterilization flows and digital tracking systems that are already in place for other instruments used in the same surgical setting. To enable change, plan phased infrastructure upgrades and incentives, by developing a “roapmap”
Regulatory barriers due to strict sterilization and tracking requirements	Same as above. In addition, collaborate with regulators to create clear protocols
More effort needed in collection and separation	Minimize need for separation and develop efficient collection and separation processes. E.g. implement matching coding systems on devices/components and collection bins
Additional maintenance and quality control	Use periodic maintenance that is already obligatory. Where possible, automate maintenance checks (e.g. in traceability systems) and enhance quality control processes
Higher risk of device malfunction	Implement robust quality assurance and monitoring
Concerns about contamination risk compared to single-use devices (leading to preference for single-use or recycling over reuse)	Design devices for easier cleanability (e.g. smooth surfaces, limited number of parts, no inaccessible cavities) and communicate safety benefits of decontamination process. Additionally, highlight that processes needed for recycling (e.g. disassembly and decontamination) already enable processes like refurbishment
Need to change whole system of manufacturers who might resist	Foster industry collaboration and incentives for system change, or make use of market differentiation business opportunities

Sustainability intervention: battery charging	
Trade-off	Potential ways to manage tension
Requires space that might not be available	Design standardized solutions for compact charging stations
Requires additional maintenance	Develop low-maintenance charging solutions

Sustainability intervention: using existing recycling systems	
Trade-off	Potential ways to manage tension
Regulatory restriction on collecting contaminated electrical devices for recycling	Ensure full decontamination before collection or explore on-site treatment options to comply with EU waste and infection control regulations

Sustainability intervention: reality-based decision-making	
Trade-off	Potential ways to manage tension
Complexity drives stakeholders to rely on assumptions rather than actual data, while those do not always reflect reality	Apply system-level thinking and sustainability assessments such as (fast-track) LCA early to guide design decisions and align perception with real impact

Sustainability intervention: radical and complex innovation	
Trade-off	Potential ways to manage tension
Preference for incremental innovation limits adoption of radical solutions	Use “transfer in perception” to frame radical innovations as approachable
Assumed infeasibility of complex alternatives without testing	Prototype early and involve end-users
Prioritization of less sustainable but simpler strategies	Promote system-level thinking and evidence-based decision making

Sustainability intervention: introducing system thinking	
Trade-off	Potential ways to manage tension
Assumed trade-offs without verifying with stakeholders	Engage diverse stakeholders in evaluation, preferably simultaneously
Financial conflicts among multiple parties	Facilitate transparent stakeholder collaboration, have all perspectives involved

Sustainability intervention: multifunctional design	
Trade-off	Potential ways to manage tension
High manufacturer costs	Base the business model and sales prices on system-level cost and savings calculations
Increased technical complexity, with high uncertainty in feasibility	Acknowledge how investment in development and standardization of modular design comes with long-term benefits
Usability concerns due to complexity	Incorporate user feedback and training (usability concerns are often highlighted by stakeholders who are not the users)
Increased inventory control and management complexity	Make use of existing logistic systems; e.g. use of instrument packs dedicated for a specific surgery
More maintenance and quality control needed	As multifunctional device replaces some other devices, need for maintenance will not necessarily increase over the full system
Weak points in device connections	Use the same connectors as used in the single functionality devices, or improve engineering and quality assurance
Coordination challenges across multiple hospital departments	Make use of existing logistic systems; e.g. use of instrument packs dedicated for a specific surgery
Difficulty balancing multifunctionality and performance	Optimize modular components for specialized tasks
Need for new storage, logistics, and tracking systems	Deploy digital tracking (medical devices are already registered for each use) and design standardized storage solutions
Higher contamination risk due to human error and misuse	Enhance training and error-proof design
Increased device cost for hospitals	Explore cost-sharing and service models or explore pay-per-use models

Sustainability intervention: enabling device traceability	
Trade-off	Potential ways to manage tension
Data privacy and protection concerns	Use secure, compliant data management systems. Do not store data inside the device
Technological challenges	Utilize the digital tracking systems that are already in place in the hospital setting
Additional regulatory compliance requirements	Collaborate with regulators early

Sustainability intervention: providing spare parts	
Trade-off	Potential ways to manage tension
Increased complexity and risk in the supply chain	Strengthen supplier networks, implement robust inventory management, and optimize logistics to ensure timely delivery of spare parts

Sustainability intervention: reducing electronics in design	
Trade-off	Potential ways to manage tension
Risk of limited adoption if perceived as lack of innovation	Communicate design rationale and benefits. Design for desired device perception
Less functionality/performance than powered variants	Aim to engineer for mechanically offering the features that powered devices offer, and if needed, integrate only part of the electronics or aim to use manual variants for low-risk surgeries only
Lower practicality in high-tech surgical environments	If certain functions cannot be offered mechanically, aim to design hybrid solutions combining manual and electronic elements
Reliance on manual input increases risk of misuse or error	Include training and fail-safes

### Sustainable design synergies

The design of medical devices for circularity is often met with resistance due to the many drawbacks highlighted previously. However, it is important to note that circular design does not always come at a cost; in some cases, it can even offer clear benefits. For example, reusable devices are not necessarily more expensive, less clinically effective, or associated with higher contamination risks—sometimes they bring savings and advantages that encourage their adoption. Similarly, during our RtD process—especially through stakeholder surveys—we identified many synergies that act as practical enablers for sustainable decision-making. The results of this analysis are summarized in Table 6.

**Table 6:** Overview of synergies

Sustainability intervention	Synergies
Use of recycled materials in production	Reduced dependence on virgin materials
Enabling safe reuse (including sterilization)	Reusing valuable components (e.g., electronics) lowers overall costs
	Reduced dependence on virgin materials
	Improved compliance with upcoming sustainability regulations
	Potential for long-term cost savings despite higher initial costs
	If properly sterilized, modular components maintain high safety standards
Multifunctional design	Consolidates instruments, reducing sterilization cycles and energy use, enhancing both circularity and cost-effectiveness
	Reduced cost per procedure
	Increase in functionality—adaptability allows for multiple surgical uses
	Potentially improved usability and user experience
	Interchangeable parts may be a first step toward standardization in the field
Radical and complex sustainable innovation	Enables market differentiation and opens business opportunities through sustainability leadership
	Built-in complexity may support close device monitoring and thus improve safety
Reducing the electronics in the design	Reduces device cost
	Easier regulatory compliance
	Compatible with existing sterilization flows (low contamination risk)
	Easy to use and learn
	Simpler technology, easier maintenance
	Better safety—no electronics in wet operating room environments
	Reduced risk of malfunction
Using existing recycling systems	Dismantling and sterilization processes needed for recycling, already enable transition toward more advanced strategies like reuse or refurbishment
	Familiarity encourages user acceptance
	Familiarity reduces the risk of improper use
Introducing system thinking	Enables development of service-based business models (e.g., product-as-a-service)
Enabling device traceability	Allows devices to be linked to patients to enable traceability in case of error

## Discussion

The results above show that redesigning laparoscopic surgical devices for circularity while improving carbon footprint is inherently challenging, given the strict demands of functionality, performance, usability, medical safety, financial viability, regulatory compliance, and technical feasibility. However, the study also suggests that these challenges are sometimes interpreted as more severe than the evidence indicates, and that considering carbon footprint and circularity can also offer important synergies, benefiting cost, safety, and clinical practice simultaneously. A clear example of this is the multifunctional device. While many stakeholders initially presented its usability as a fundamental trade-off—arguing that surgeons would find such a device impractical—the surgeon participating in the study ultimately selected it as his preferred option, precisely citing its practicality and ease of use, which contrasted with assumptions voiced by others. This discrepancy may illustrate how interpretations of barriers, including compliance requirements and waste definitions, can obscure real opportunities for innovation. Similar patterns have been noted in previous work, where barriers to sustainable medical device design were often more perceptual than factual, particularly regarding safety and regulatory compliance (Hoveling et al., 2024).

Beyond individual perceptions of usability, comparing the LCA results with stakeholder and expert evaluations greatly changed the final design direction. The comparison showed that environmental performance alone did not determine concept preference: concepts with higher perceived feasibility were not necessarily those with the greatest carbon reduction potential. For example, the recyclable device was preferred by Medtech experts because it represented the “most advanced yet acceptable” option—closest to current devices in use and product architecture, and therefore easiest to integrate into existing clinical workflows and regulatory pathways. However, it provided the least carbon reduction, because the main manufacturing hotspots remained unchanged. Conversely, the multifunctional and manual concepts showed greater environmental benefit but lacked technical feasibility or clinical performance. This made the recyclable device a pragmatic first step toward circularity, enabling faster adoption and more immediate impact compared to more radical redesigns. In contrast, other stakeholders rejected the recyclable device precisely because they perceived it as environmentally inferior. Instead, they preferred concepts such as the partially reusable and multifunctional devices, which they associated with stronger environmental performance and practical synergies. Overall, stakeholder preferences showed no consistent alignment with the concepts showing the highest estimated environmental improvements, with only half of participants selecting concepts associated with clear LCA advantages. This mismatch underscores a central challenge of circular medical device design: sustainability, technical criteria, clinical usability, and other criteria are separate filters on design ideas, so the best overall design rarely maximizes any single criterion. Companies decide which criteria to prioritize, and in healthcare, patient safety and risk management are critical. But to make informed decisions, companies should model the environmental consequences of their choices just as they already model the financial and clinical consequences. Even when the most sustainable design option is not chosen, it can point the way toward future innovation, helping ideas lead from today’s incremental strategies that enable fast adoption and scaling, toward more transformative circular redesigns that could offer much greater environmental benefits but require more radical changes to established product architectures and/or clinical, regulatory, or business practices. Making evidence-based decisions like this, with LCA models alongside models of cost, clinical effectiveness, and other factors, can inform not just manufacturer’s product development, but also hospital procurement policies and government regulatory frameworks.

At the same time, the findings show that circular strategies can yield unexpected synergies rather than trade-offs. Enabling reuse and sterilization not only reduces reliance on virgin materials but can also lower costs by extending the life of high-value components, improve alignment with emerging regulations, and generate long-term savings despite higher upfront investment. Similarly, reducing or eliminating electronic components simplifies production, can reduce upfront costs and potentially reduce long-term costs through simpler maintenance and reduced failure risks, enhances sterilization compatibility, may reduce risks associated with electronic failure in operating room environments, reduces malfunctioning, and can make devices more intuitive for staff. These findings suggest that circular design, when done thoughtfully, does not always involve sacrifice—it may in fact lead to multi-dimensional improvements.

These observations also reinforce that achieving circularity in medical device design is not solely a technical challenge but a systems-level one. Successful implementation requires integrated strategies that align design innovation with regulatory frameworks, clinical practices, supply chain logistics, and economic incentives across the healthcare ecosystem. This aligns with broader evidence: Hossain et al., (2025) emphasize the importance of integrating circular strategies with healthcare system dynamics; Kane et al., (2018) highlight the necessity of considering hygiene criticality and sterilization processes in device design; and Hoveling et al., (2024) identify systemic barriers such as regulatory constraints and stakeholder dynamics that hinder circular implementation.

From this systems perspective, the findings also have implications for implementation, including procurement strategies and business model development. Circular medical devices may require procurement approaches that consider lifecycle value rather than only upfront purchase costs. Furthermore, reuse-oriented strategies require alignment with regulatory pathways, waste management systems, and traceability requirements. For example, regulatory frameworks such as the European Medical Device Regulation (MDR) and waste legislation such as the Waste Electrical and Electronic Equipment (WEEE) Directive may influence requirements related to device traceability, reuse validation, material recovery, and end-of-life management. Business models such as product-as-a-service or pay-per-use approaches may provide mechanisms to distribute costs and incentives across manufacturers and healthcare providers.

Taken together, these insights underscore the need for design processes that integrate both quantitative assessments (e.g., whole system LCA) and qualitative stakeholder perspectives. The integrated assessment used in this study demonstrates that combining environmental performance with stakeholder evaluation helps to find tradeoffs and/or synergies. Focusing solely on technical solutions or environmental metrics, as much previous research has done, is insufficient. Instead, structured design approaches—such as the one demonstrated in this study—can surface synergies, distinguish factual from perceptual trade-offs, and generate actionable recommendations that advance sustainability while respecting clinical and regulatory realities. Although the specific redesign concepts and environmental outcomes identified here are specific to powered laparoscopic staplers, the underlying approach is transferable to other medical devices, particularly electronics-intensive devices and products operating within highly regulated environments. The value of this case study therefore lies not only in the proposed redesigns, but in demonstrating a process for evidence-based decision-making when balancing sustainability goals with clinical, regulatory, and business requirements. Specifically, design strategies directly related to stapler functionality, surgical workflow, and laparoscopic procedures remain context-specific. In contrast, broader principles—such as assessing environmental hotspots early, integrating stakeholder perspectives, and identifying trade-offs between circularity and regulatory or operational requirements—are applicable to other complex medical devices.

### Limitations

This study examined a single case of one laparoscopic device. While this enabled an in-depth analysis of trade-offs, the narrow scope limits the generalizability of findings to the broader medical device sector. Additional studies across diverse device types and clinical contexts are needed to validate and extend these insights. Moreover, the assessed products and systems were conceptual designs, meaning the results depend on assumptions and interpretations that may differ in real-world implementation. Since no physical prototypes were developed, stakeholder evaluations were based on the perceived feasibility, usability, and desirability of the proposed concepts rather than observed performance in clinical settings. Furthermore, differences in stakeholder familiarity with circular design principles and medical device development may have influenced how concepts were interpreted. Therefore, expert and survey-based evaluations may be influenced by differences in individual experience, interpretation of the concepts, and the level of detail provided. Future research should validate these findings through prototyping, user testing, and experimental evaluation of technical performance, usability, and sterilization feasibility. Additionally, applying the proposed design process to other medical device categories will help determine which trade-offs are specific to laparoscopic staplers and which represent broader challenges in circular medical device design.

Similarly, the fast-track LCA approach involves uncertainty due to the early-stage nature of the concepts and the limited availability of detailed product-specific data. In particular, assumptions regarding reuse cycles, sterilization requirements, maintenance frequency, and material choices may influence the absolute environmental impacts. These parameters represent important uncertainty drivers that should be explored through sensitivity analyses once validated product and process data become available. Therefore, the LCA results should be interpreted primarily as a comparative assessment of the relative potential of circular design strategies rather than as precise predictions of future carbon footprints.

The stakeholders interviewed were a limited sample (N = 10) for qualitative feedback, not quantitative ranking; they were selected to represent different stakeholder groups involved in circular medical device design, to represent clinical, sustainability, technical, and business perspectives. This purposive sampling supported a systems-level evaluation of circular design concepts but did not allow differences between stakeholder groups to be analyzed separately. Consequently, the stakeholder findings should be interpreted as exploratory insights into potential trade-offs and synergies rather than as evidence of consensus across the healthcare sector. Furthermore, MedTech experts evaluated the concepts using a 1–4 scoring system, while survey participants used a 1–5 Likert scale. This difference was necessary to enable direct comparison between concepts for experts, but it also posed a limitation because experts could not assign equal scores to multiple concepts, whereas survey participants could. The central contribution is to demonstrate how integrating quantitative assessments (e.g., LCA) with stakeholder input can help stakeholders make more evidence-based decisions, guiding circular innovation and uncover synergies between sustainability and operational requirements. Even a single-case analysis, as shown here, can highlight key trade-offs, potential synergies, and considerations relevant to advancing circularity in medical devices.

### Future research

Future research should build on these findings by developing a circular design guide tailored to medical devices. This guide should provide a structured, step-by-step overview of the product development process with an embedded mapping of common trade-offs at each phase, prompting decisions based on evidence (e.g., LCA and user tests) rather than assumptions. It should also identify strategies to positively address or mitigate those trade-offs. Furthermore, research should explore how these strategies differ by device type, clinical use case, and geographical/regulatory context, and examine how early-stage collaboration between stakeholders can lead to more holistic and effective outcomes. Future RtD iterations could further refine the concepts by directly addressing the trade-offs identified in this study. For example, economic barriers associated with recyclable designs could be explored through alternative take-back or producer-responsibility models, while reuse-related concerns could be investigated through service-based approaches where specialized parties manage sterilization and maintenance. Such iterative refinement could help translate promising circular design strategies into more implementation-ready solutions.

## Conclusion

This study explored how trade-offs and synergies between circularity and carbon footprint and safety, functionality, usability, and regulatory compliance can be assessed and managed in medical device design. Our findings uncovered several important trade-offs, such as additional sterilization steps, increased technical complexity, or workflow adjustments. However, even under strict clinical and regulatory constraints, those trade-offs can be managed or mitigated, despite there not being many examples of this in practice. Simultaneously, leverageable synergies were identified, where circular strategies improve both environmental and operational outcomes. Comparing a baseline single-use laparoscopic stapler with redesigned concepts, multifunctional and manual designs could theoretically reduce environmental impacts by 80–90% but realizing these gains in practice remains challenging. Partially reusable devices offer moderate, feasible improvements, and fully recyclable single-use designs provide limited benefits, though it might be easier to implement. Stakeholder evaluations suggested that technical feasibility, usability, and workflow constraints often influence design decisions more than environmental limitations. While these constraints may not be regulatory, failing to address them can lead to complicated device use and subsequent issues. Regulatory compliance therefore represents the minimum requirement; building beyond it improves device performance and outcomes. Integrating environmental assessment with stakeholder insights proved essential to distinguish factual trade-offs from assumptions, and to identify opportunities where circular strategies can enhance safety, usability, or cost-efficiency. While some of the most circular strategies remain difficult to implement under current conditions, whole system approaches integrating LCA models with clinical and financial models can guide future goals for circular design in healthcare. When approached thoughtfully, it can deliver multidimensional benefits, supporting patients, clinicians, industry, and the environment alike.

## Summary:

### Supplementary Information SI1:

This supporting information provides supplementary materials supporting the study methodology and results, including the simplified product journey map, relevant standards and regulations, circular design requirements, stakeholder survey materials, participant characteristics, detailed LCA assumptions, and stakeholder and Medtech expert evaluation scores.

## Supplementary Information

Below is the link to the electronic supplementary material.

Supplementary file1 (DOCX 3353 KB)

## Data Availability

Interview and survey data are not publicly available due to privacy and ethical restrictions. Life cycle assessment (LCA) data are subject to third-party restrictions.

## Footnotes

1 **Aseptic transfer** refers to the process of moving materials without contamination, maintaining sterility to ensure safety and prevent infection in medical settings.
