# Supplementary material for: Balancing trade-offs in circular medical device design: a case study on laparoscopic devices
Source: J Ind Ecol. 2026 Sep 20;30(5):2697–713. doi: 10.1007/s44498-026-00189-8 (PMC13649933; doi:10.1007/s44498-026-00189-8)
Supplement: Supplementary file 1 [file 44498_2026_189_MOESM1_ESM.docx]

SUPPORTING INFORMATION FOR:

Hoveling, T., Muindi, N., Faludi, J. & Bakker, C. Balancing Trade-Offs in Circular Medical Device Design: A Case Study on Laparoscopic Devices. *Journal of Industrial Ecology.*

This supporting information provides supplementary materials supporting the study methodology and results, including the simplified product journey map, relevant standards and regulations, circular design requirements, stakeholder survey materials, participant characteristics, detailed LCA assumptions, and stakeholder and Medtech expert evaluation scores.

*Tables in the supporting information with more than 50 numerical data points (e.g., data tables with 5 columns and 10 rows) should be provided in a numerical format (e.g., comma-separated value files or a spreadsheet). For a template for spreadsheets used as supporting information, please visit* [*http://jie.click/templates*](http://jie.click/templates)*.*

**APPENDIX A: VISUALISATION OF LAPAROSCOPY**

Figure 3: Laparoscopic Surgery

# APPENDIX B: SIMPLIFIED VERSION OF PRODUCT JOURNEY MAP

Figure 4: Product Journey Map (simplified version)

# APPENDIX C: Standards and regulations research

Surgical staplers adhere to rigorous standards and regulations. These standards ensure not only the safety and effectiveness of the device but also align with increasing demands for environmental sustainability. As the healthcare industry faces mounting pressure to reduce its environmental footprint, integrating circular economy principles into medical device design has become imperative. This chapter provides an overview of the applicable standards and regulations for laparoscopic devices, categorized into general product-specific and environmentally focused guidelines. Additionally, it outlines corresponding design guidelines, identifying overlaps and potential contradictions, thus offering a holistic perspective on the challenges and opportunities in designing sustainable laparoscopic devices.

## General Standards and Regulations

The following general standards and regulations apply to the design and production of laparoscopic devices:

- EU Medical Device Regulation (MDR) 2017/745
- ISO 13485:2016 - Quality Management Systems for Medical Devices
- ISO 14971:2019 - Risk Management for Medical Devices
- IEC 60601 Series - Medical Electrical Equipment
  - IEC 60601-1: General safety and performance.
  - IEC 60601-1-2: Electromagnetic compatibility.
  - IEC 60601-2-2: High-frequency surgical equipment.
- ISO 11135:2014 - Sterilization of Health Care Products
- ISO 11607 Series - Packaging for Terminally Sterilized Medical Devices
- ISO 17664:2017 - Processing of Health Care Products
- ISO 10993 Series - Biological Evaluation of Medical Devices
  - ISO 10993-1: Biocompatibility testing.
- IEC 62366-1:2015 - Usability Engineering for Medical Devices
- ISO 14155:2020 - Clinical Investigation of Medical Devices for Human Subjects
- ISO 11737 Series - Sterilization of Health Care Products
- FDA Regulations (if applicable)
  - 21 CFR Part 820 - Quality System Regulation (QSR)
  - 21 CFR Part 807 - Establishment Registration and Device Listing
  - 21 CFR Part 814 - Premarket Approval (PMA)
  - 21 CFR Part 803 - Medical Device Reporting (MDR)
- Medical Device Single Audit Program (MDSAP)

### General Product-Specific Design Guidelines

Based on the general standards and regulations listed, we propose the following design guidelines to be considered in the redesign of the laparoscopic device:

1. **Safety and Performance**: Design must prioritize patient safety, efficacy, and compliance with international safety standards (ISO 13485, IEC 60601 series, MDR).
2. **Usability**: Ensure user-friendly interface and ergonomics to minimize user errors and enhance clinical outcomes (IEC 62366-1).
3. **Sterilization Compatibility**: Must be compatible with standard sterilization methods (ISO 11135, ISO 17664).
4. **Biocompatibility**: Use materials that are biocompatible and suitable for prolonged patient contact (ISO 10993 series).
5. **Quality Management**: Implement robust quality management systems (ISO 13485, FDA QSR).
6. **Risk Management**: Thoroughly assess and mitigate risks associated with device use (ISO 14971).
7. **Clinical Evaluation**: Conduct comprehensive clinical evaluations and trials to validate safety and effectiveness (ISO 14155).
8. **Packaging**: Ensure sterile barrier and mechanical protection during transport and storage (ISO 11607 series).

## Environmental Sustainability Related Standards and Regulations

The full list of environmental sustainability related standards and regulations we identified to be applicable to the laparoscopic device are listed below:

1. Eco-design for Sustainable Products Regulation (ESPR)
2. EU Directive 2012/19/EU - Waste Electrical and Electronic Equipment (WEEE) Directive
3. EU Directive 2011/65/EU - Restriction of Hazardous Substances (RoHS) Directive
4. EU Packaging and Packaging Waste Directive (PPWD)
5. EU Directive 2008/98/EC - Waste Framework Directive (WFD)
6. ISO 14001:2015 - Environmental Management Systems
7. ISO 14040:2006 - Life Cycle Assessment (LCA) Principles and Framework
8. REACH Regulation (EC) No 1907/2006
9. Right to Repair Legislation (EC 2023)
10. ISO 14025:2006 - Environmental Labels and Declarations
11. ISO 20400:2017 - Sustainable Procurement
12. ISO 59004:2024 - Circular Economy — Vocabulary, Principles, and Guidance for Implementation
13. California Proposition 65 (if applicable)
14. EU Battery Regulation (2025)

### Environmental Sustainability Design Guidelines

1. **Resource Efficiency**: Minimize material use and select environmentally friendly materials, including recycled or recyclable content (ESPR, ISO 14001).
2. **Energy Efficiency**: Optimize energy use during manufacturing and, if applicable, during use (ESPR).
3. **Hazardous Substance Management**: Avoid or minimize hazardous substances in materials and components (RoHS Directive, REACH).
4. **Design for Disassembly**: Facilitate easy disassembly for repair, recycling, or safe disposal (Right to Repair).
5. **EoL Management**: Plan for efficient recycling and safe disposal, including compliance with WEEE and PPWD (WEEE Directive, PPWD).
6. **Eco-Labeling and Transparency**: Provide clear information on environmental impact, recyclability, and proper disposal methods (ISO 14025).
7. **Life Cycle Assessment**: Conduct LCAs to understand and minimize environmental impacts throughout the product lifecycle (ISO 14040).
8. **Sustainable Procurement**: Prioritize suppliers and materials that adhere to sustainability standards (ISO 20400).

## Overlap and Contradictions

Balancing general product requirements with environmental sustainability guidelines can present both opportunities and challenges. Below, we explore the areas of overlap and potential contradictions between these two sets of design requirements.

### Overlap

- **Materials Selection**: Both lists emphasize selecting biocompatible and non-toxic materials that are safe for patients and the environment.
- **Risk Management**: Both require a comprehensive assessment of risks, whether for patient safety (ISO 14971) or environmental impact (ISO 14001).
- **Packaging**: While the general guidelines focus on maintaining sterility and safety, sustainability guidelines emphasize minimizing environmental impact through recyclable packaging materials.

### Contradictions

- **Design for Durability vs. Disassembly**: General guidelines often prioritize durable and robust designs for safety and long-term use. In contrast, sustainability guidelines may require designs that facilitate easy disassembly for recycling or repair, which could sometimes compromise the structural integrity and durability of the device.
- **Single use vs. Reusability**: General medical device standards may necessitate single use devices for hygiene and safety, while sustainability standards advocate for reusable components or devices to reduce waste.
- **Use of recycled materials**: While recycling is favourable in circular economy contexts, ISO 11607 recommends against this (even for primary packaging), and advocates for full traceability of such materials: which current technologies, it is therefore unlikely that anything other than virgin manufacturing will be used in recycled materials, due to insufficient controls to allow the safe use of other recycled material in sterile barrier systems.

# APPENDIX D: DESIGN REQUIREMENTS

Designing laparoscopic staplers that are both effective and environmentally sustainable presents unique challenges and opportunities. This chapter outlines key design considerations derived from previous research (Hoveling et al., 2024), industry standards and regulations (Appendix C), and prior public as well as confidential deliverables from the Digital Health in the Circular Economy (DiCE) project (*Deliverables & Reports - Digital Health in the Circular Economy*, 2023). Additional non-circular requirements we added based on the performed OR observations. The goal is to provide a framework that balances high performance with environmental responsibility, ensuring that laparoscopic devices meet rigorous quality, safety, and usability standards while adhering to sustainable practices. Therefore, the final solidified list of design guidelines for the laparoscopic stapler is presented in the seven categories below, combining circular and non-circular requirements rather than separating them.

It is important to note that while a full list of all relevant design requirements is presented here, not all requirements are a priority in the redesign. For example, while requirement 7.1 suggests optimizing energy consumption during use as our earlier research suggests this can benefit circularity (Hoveling et al., 2024), this requirement should not be prioritized as the LCA results suggest that the use phase is not an impact hotspot for this specific product. However, this list also exemplifies what an integrated list of circular and non-circular design requirements could looks like for a laparoscopic device, rather than only serving as input for this specific redesign task.

## Quality & Performance

1.1 In the redesign, maintain or improve the quality, function, and usability of the laparoscopic device compared to previous models.
1.2 Ensure reliable performance in insertion, tissue grabbing, stapling, and cutting functions.
1.3 Design the laparoscopic device to handle various types and thicknesses of tissue through interchangeable cartridges and staples.
1.4 Maximize the use of all functionalities of the laparoscopic device to enhance perceived value and reduce early disposal.
1.5 Comply with FDA regulations (if applicable), including 21 CFR Part 820 and 21 CFR Part 814, while also adhering to ISO 13485:2016 and MDR 2017/745.

## Patient Safety

2.1 Design the laparoscopic device to close tissue properly without leakage, ensuring effective closure during the stapling process.
2.2 Ensure stable operation to prevent instability that could cause damage or compromise the effectiveness of the device.
2.3 Maintain sterility through its entire use phase, complying with ISO 11135:2014 and ISO 11607 Series for sterilization and packaging.
2.4 Conduct thorough risk assessments and implement measures to mitigate risks associated with device use, as outlined in ISO 14971:2019.

## Usability & Ergonomics

3.1 Design the laparoscopic device to be easily handled by users with varying hand sizes and strengths, with an appropriately sized handle and minimal force required for operation.
3.2 Ensure the laparoscopic device is suitable for use in various healthcare settings, facilitating easy transport and handling.
3.3 Provide clear and user-friendly instructions for use, maintenance, and disposal.
3.4 Incorporate features that facilitate troubleshooting and repair.

## Manufacturing & Production

4.1 Ensure the laparoscopic device can be mass-produced using standardized techniques to maintain cost-effectiveness and scalability.
4.2 Enable faster production adjustments and support by localizing manufacturing and distribution.
4.3 Design the laparoscopic device to use minimal materials while maintaining quality and functionality. Accommodate various production methods beyond injection moulding.

## Durability & Maintenance

5.1 Ensure materials and components are durable enough to withstand necessary chemicals, temperatures, handling, UV exposure, and liquids.
5.2 Design the laparoscopic device to tolerate exposure to various decontamination methods, especially the needed heat sterilization.
5.3 Incorporate smooth surfaces and minimize hard-to-reach areas to prevent dirt and microbe accumulation.
5.4 Provide clear guidelines for authorized parties on cleaning, disinfecting, and decontaminating the product. Ensure accessible product use history information for quality checks and decontamination.

## Information & Traceability

6.1 Provide a digital product passport detailing materials, sustainability, and recyclability, in line with **ESPR** and **ISO 14025:2006**.
6.2 Implement a system to track and record the number of use cycles and unique device identification for inventory management and tracking.

## Materials & Components

7.1 Minimize material use and select environmentally friendly materials, including recycled or recyclable content.
7.2 Minimize the use of critical raw materials, such as tungsten and cobalt, where possible. Opt for alternative materials to mitigate supply chain risks and adhere to the **EU Critical Raw Materials Regulation**.
7.3 Optimize energy consumption during manufacturing and use.
7.4 Comply with chemical restrictions environmental legislations (EU REACH Restriction, Stockholm convention on persistent organic pollutants, EU Restriction on Hazardous Substances in EEE & EU Battery Regulation) and avoid other substances of concern (EU REACH SVHC and similar).
7.5 Design for easy disassembly to facilitate repair, recycling, or safe disposal. Ensure compliance with **Right to Repair legislation** and **WEEE Directive (2012/19/EU)**.
7.6 Avoid black plastics due to sorting difficulties and select polymers with densities of less than 1.1 g/cm³.
7.7 Use packaging materials that are recyclable and reusable, with minimal hazardous substances (comply with requirements under EU PPWR).
7.8 Provide clear labelling on packaging to facilitate recycling.

**APPENDIX E: PARTICIPANT CHARACTERISTICS**

Table 1: List of survey participants

| **P#** | **Job title** | **Expertise** | **Healthcare experience** |
| --- | --- | --- | --- |
| **1** | General Surgeon | Surgery execution and testing new laparoscopic products | Yes |
| **2** | Sustainability Business Analyst | Sustainability, industrial design | Yes |
| **3** | Assistant Professor | Sustainable design | Yes |
| **4** | Technical Advisor | Collection, reverse logistics, recycling, WEEE take-back schemes | No |
| **5** | Chief Business Development Officer | Smart collection systems | No |
| **6** | Project Management | Circular business models | No |
| **7** | Research Associate | Digital Nudging, AI, Sustainability | No |
| **8** | Senior Manager in Business Transformation & Innovation | Advanced technologies and innovation | Yes |
| **9** | Business Developer | Healthcare industry (pharma & medical devices), business models, behaviour strategies | Yes |
| **10** | Assistant Sustainability Project Manager | Project management, sustainability, carbon footprinting | Yes |

# APPENDIX F: STAKEHOLDER SURVEY

**Consent form**
*_________________________________________________________________________________________________________________________________*
 **Dear Participant,**

Thank you for participating in this research, led by PhD candidate Tamara Hoveling from TU Delft as part of the Horizon Europe-funded DiCE project, which aims to reduce e-waste from health devices. This study focuses on the redesign of a single use endocutter (linear stapler). In this document, each of four conceptual redesigns developed by TU Delft will be explained separately, each followed by a short survey for your feedback. In some cases, a brief call may be scheduled to discuss your answers in detail.

**By signing this form, you confirm that you are informed about the research, voluntarily agree to participate, and consent to the use of your anonymized answers in scientific publications. Participation is entirely voluntary, and you may withdraw at any time without reason. Please feel free to ask any questions. Note that data cannot be removed once your answers have been shared with the researcher.**

**Contact**: Tamara Hoveling, [t.hoveling@tudelft.nl](mailto:t.hoveling@tudelft.nl)

**Name:	Date:**

**Do you agree to participate in this research?**	☒ yes	☐ no


**After completing the survey, please sign below.**

*Name is needed for giving consent only.
Your answers in this survey will be handled confidentially and will be published anonymously*

**First, please answer some general questions**

1. **What is your current job title?**
2. **Please state your expertise(s) below**

***(For example, recycling, medical safety, business models, supply chain management)***

3. **Years of experience in this expertise**

4. **Do you have experience in the healthcare industry?**

☐ yes	☐ no

**EXPLANATION FOR CONCEPT A –** FULLY RECYCLABLE DEVICE

The first concept is a fully recyclable device. The linear stapler is recycled directly after one use. In this process, the hospital separates the battery from the device, decontaminates the components, and sells both the battery and the device to recycling companies. At the recycling facility, materials such as ferrous and non-ferrous metals, as well as high- and low-value plastics, are separated and recycled for use in the production of new devices. Since this concept assumes the highest safety standards, it also presumes the use of virgin materials for production rather than recycled inputs.

The device is compact and designed with a "reduce" approach, using minimal materials. It consists of three click-separable compartments: a durable metal shaft made from recyclable surgical stainless steel, an injection-moulded plastic casing of sterilizable medical-grade polypropylene, and an electronics unit with a separable single-use battery. Clear icons guide users through a simple disassembly process to enable efficient recycling. For clarification, refer to Figure 5 (simplified system map) and Figure 6 (product design sketches).

*Figure 5: Simplified system map concept A*

*Figure 6: Product design sketches concept A*

**SURVEY FOR CONCEPT A –** FULLY RECYCLABLE DEVICE

2. What are your general thoughts about this concept?
1. For each perspective below, please indicate how you would rate each perspective below for the just-presented design concept. Check the box that best represents your assessment.

*We understand that some of these perspectives may involve uncertainties requiring further research. Please feel free to base your answers on your professional judgment and experience—your estimations are valuable and will help guide future development*.

| *Perspective to rate …* | Very poor | Poor | Acceptable | Good | Very good |
| --- | --- | --- | --- | --- | --- |
| **Environmental sustainability** | ☐ | ☐ | ☐ | ☐ | ☐ |
| *Type here to explain answer* | | | | | |
| **Financial viability** | ☐ | ☐ | ☐ | ☐ | ☐ |
| *Type here to explain answer* | | | | | |
| **Logistic feasibility** | ☐ | ☐ | ☐ | ☐ | ☐ |
| *Type here to explain answer* | | | | | |
| **Functionality and performance** | ☐ | ☐ | ☐ | ☐ | ☐ |
| *Type here to explain answer* | | | | | |
| **Ease of use and maintenance** | ☐ | ☐ | ☐ | ☐ | ☐ |
| *Type here to explain answer* | | | | | |
| **Regulatory compliance** | ☐ | ☐ | ☐ | ☐ | ☐ |
| *Type here to explain answer* | | | | | |
| **Technical feasibility** | ☐ | ☐ | ☐ | ☐ | ☐ |
| *Type here to explain answer* | | | | | |

**EXPLANATION FOR CONCEPT B –** PARTIALLY REUSABLE DEVICE

The second concept is a partially reusable device. Unlike the fully recyclable model, this design allows the internal electronics unit to be reused while the outer components remain single-use and recyclable. After each procedure, hospital staff removes the electronics unit for recharging, while the sterile plastic casing and other disposable parts are sent for recycling. The device consists of three click-separable compartments: a durable metal shaft, a single-use sterile plastic casing, and a reusable electronics unit.

This design reduces electronic waste while ensuring a safe and efficient workflow. Clear icons guide hospital staff through the separation process, making disassembly straightforward. After approximately 200 uses, the reusable electronics unit is also recycled. By minimizing single-use electronic waste while maintaining high safety standards, this concept strikes a balance between sustainability and practicality. For clarification, refer to Figure 7 (simplified system map) and Figure 8 (product design sketches).

*Figure 7: Simplified system map concept B*

*Figure 8: Product design sketches concept B*

**SURVEY FOR CONCEPT B –** PARTIALLY REUSABLE DEVICE

*Figure 8: Product design sketches concept B*

**SURVEY FOR CONCEPT B –** PARTIALLY REUSABLE DEVICE

3. What are your general thoughts about this concept?
2. For each perspective below, please indicate how you would rate each perspective below for the just-presented design concept. Check the box that best represents your assessment.

*We understand that some of these perspectives may involve uncertainties requiring further research. Please feel free to base your answers on your professional judgment and experience—your estimations are valuable and will help guide future development*.

| *Perspective to rate …* | Very poor | Poor | Acceptable | Good | Very good |
| --- | --- | --- | --- | --- | --- |
| **Environmental sustainability** | ☒ | ☐ | ☐ | ☐ | ☐ |
| *Type here to explain answer* | | | | | |
| **Financial viability** | ☐ | ☐ | ☐ | ☐ | ☐ |
| *Type here to explain answer* | | | | | |
| **Logistic feasibility** | ☐ | ☐ | ☐ | ☐ | ☐ |
| *Type here to explain answer* | | | | | |
| **Functionality and performance** | ☐ | ☐ | ☐ | ☐ | ☐ |
| *Type here to explain answer* | | | | | |
| **Ease of use and maintenance** | ☐ | ☐ | ☐ | ☐ | ☐ |
| *Type here to explain answer* | | | | | |
| **Regulatory compliance** | ☐ | ☐ | ☐ | ☐ | ☐ |
| *Type here to explain answer* | | | | | |
| **Technical feasibility** | ☐ | ☐ | ☐ | ☐ | ☐ |
| *Type here to explain answer* | | | | | |

**EXPLANATION FOR CONCEPT C –** MULTIFUNCTIONAL DEVICE

The third concept is a modular, multifunctional device with a reusable main unit and interchangeable click-on shafts for different surgical tasks. The main unit houses an electronics module, while the shafts and outer shell can be swapped depending on the procedure. After each use, the shafts and shell are sterilized, while the electronics unit is wiped down and recharged. A tracking system monitors usage, ensuring parts are replaced when they reach their limit and sent for recycling or refurbishment.

This design extends the device’s lifespan, reduces waste, and offers flexibility for different surgeries. The click-on shafts are color-coded for easy identification and can also be used with robotic surgical arms. Once the electronics unit reaches its maximum charge cycles, it is returned to the manufacturer for inspection and potential refurbishment. If reuse is not possible, it is recycled. For clarification, refer to Figure 9 (simplified system map) and Figure 10 (product design sketches).

Figure **9**: Simplified system map concept C

*Figure 10: Product design sketches concept C*

**SURVEY FOR CONCEPT C –** MULTIFUNCTIONAL DEVICE

4. What are your general thoughts about this concept?
3. For each perspective below, please indicate how you would rate each perspective below for the just-presented design concept. Check the box that best represents your assessment.

*We understand that some of these perspectives may involve uncertainties requiring further research. Please feel free to base your answers on your professional judgment and experience—your estimations are valuable and will help guide future development*.

| *Perspective to rate …* | Very poor | Poor | Acceptable | Good | Very good |
| --- | --- | --- | --- | --- | --- |
| **Environmental sustainability** | ☐ | ☐ | ☐ | ☐ | ☐ |
| *Type here to explain answer* | | | | | |
| **Financial viability** | ☐ | ☐ | ☐ | ☐ | ☐ |
| *Type here to explain answer* | | | | | |
| **Logistic feasibility** | ☐ | ☐ | ☐ | ☐ | ☐ |
| *Type here to explain answer* | | | | | |
| **Functionality and performance** | ☐ | ☐ | ☐ | ☐ | ☐ |
| *Type here to explain answer* | | | | | |
| **Ease of use and maintenance** | ☐ | ☐ | ☐ | ☐ | ☐ |
| *Type here to explain answer* | | | | | |
| **Regulatory compliance** | ☐ | ☐ | ☐ | ☐ | ☐ |
| *Type here to explain answer* | | | | | |
| **Technical feasibility** | ☐ | ☐ | ☐ | ☐ | ☐ |
| *Type here to explain answer* | | | | | |

**EXPLANATION FOR CONCEPT D –** MANUAL DEVICE

The fourth concept is a manual device designed for simplicity and reuse, focusing on minimizing reliance on electronics. This device is intended for full reuse without the need for complex separation or logistics in the operating room. After each use, the device is ready for cleaning and sterilization. Once it reaches the maximum number of reuse cycles, it is refurbished. When refurbishment is no longer possible, the device is then recycled. While the environmental impact of recycling and refurbishment may be minimal due to the lack of electronics, the focus is on creating a high-value, durable device.

As this concept explores a device entirely driven by manual force, the initial design approach focused on gathering insights and feedback from experts regarding the feasibility of a manual-driven device. Therefore, no detailed product sketches or storyboards were developed at this stage. This early stage aimed to assess whether a purely manual device could meet user needs before refining it further with specific technical details. For clarification of this concept, refer to Figure 11 (simplified system map).

*Figure 11: Simplified system map concept D*

**SURVEY FOR CONCEPT D –** MANUAL DEVICE

5. What are your general thoughts about this concept?
4. For each perspective below, please indicate how you would rate each perspective below for the just-presented design concept. Check the box that best represents your assessment.

*We understand that some of these perspectives may involve uncertainties requiring further research. Please feel free to base your answers on your professional judgment and experience—your estimations are valuable and will help guide future development*.

| *Perspective to rate …* | Very poor | Poor | Acceptable | Good | Very good |
| --- | --- | --- | --- | --- | --- |
| **Environmental sustainability** | ☐ | ☐ | ☐ | ☐ | ☐ |
| *Type here to explain answer* | | | | | |
| **Financial viability** | ☐ | ☐ | ☐ | ☐ | ☐ |
| *Type here to explain answer* | | | | | |
| **Logistic feasibility** | ☐ | ☐ | ☐ | ☐ | ☐ |
| *Type here to explain answer* | | | | | |
| **Functionality and performance** | ☐ | ☐ | ☐ | ☐ | ☐ |
| *Type here to explain answer* | | | | | |
| **Ease of use and maintenance** | ☐ | ☐ | ☐ | ☐ | ☐ |
| *Type here to explain answer* | | | | | |
| **Regulatory compliance** | ☐ | ☐ | ☐ | ☐ | ☐ |
| *Type here to explain answer* | | | | | |
| **Technical feasibility** | ☐ | ☐ | ☐ | ☐ | ☐ |
| *Type here to explain answer* | | | | | |

**FINAL QUESTIONS – CONCEPT COMPARISON**

*Thank you for your feedback on our concepts! To finalize this survey, please fill-in these final questions for comparison.*

1. **Off all four concepts, which one would be your overall favourite?**

☐ A – Fully Recyclable Device

☐ B – Partially Reusable Device

☐ C – Multifunctional Device

☐ D – Manual Device

2. **Why did you choose this concept?**

3. **Below, add any additional thoughts you’d like to share with us.**

**THANK YOU FOR COMPLETING THIS SURVEY!**

**PLEASE DO NOT FORGET TO SIGN THE CONSENT FORM ON PAGE ONE**

# **APPENDIX G: OUTCOME OF THE BASELINE LCA**

Figure 12: Summarized results of baseline LCA. Climate change in kg CO2-eq per process (50 use cycles, and climate change of production per material type per device (in “production” bar). Gradients represent uncertainties. The underlying LCA data used to generate this figure are not publicly available due to third-party confidentiality restrictions; see the Data Availability Statement.

**APPENDIX H: LCA ASSUMPTIONS**

Table 7: LCA assumptions per design concept

| **Design Concept** | **Life cycle assumptions made for LCA calculations *(compared to baseline)*** |
| --- | --- |
| **General changes assumed for all concepts** | - Metals → 316L stainless steel: preserves strength/biocompatibility - Plastics → medical-grade PP: adequate strength, may slightly alter weight/balance - Functional equivalence maintained: all concepts meet standard surgical performance (staple line integrity, compression, trigger reliability, laparoscopic workflow compatibility) |
| **Recyclable Device** | - General changes (as above) - Full device sterilized and recycled after each use |
| **Partially Reusable Device** | - General changes (as above) - Reused electronics unit contains battery and PCB - Remaining plastics/metals sterilized and recycled - Split-use sterilization: patient-contact parts single-use; non-sterile components disinfected with IPA wipes - Battery replaced by a rechargeable battery |
| **Multifunctional Device** | - General changes (as above) - Sequential functions only; energy sealing excluded - Test was performed based on functionalities used in common surgeries - Minor learning curve possible; ergonomics acceptable |
| **Manual Device** | - General changes (as above) - No electronics; fully mechanical - Device fully reusable and sterilizable - All components go to recycling at EoL |
